# Happiness and Achievement Motivation among Iranian Nursing Students: A Descriptive Correlational Study

**DOI:** 10.1155/2022/4007048

**Published:** 2022-04-20

**Authors:** Jamileh Amani Nezhad, Farokh Abazari, Abbas Mardani, Maryam Maleki, Toni Hebda

**Affiliations:** ^1^Department of Community Health, Nursing and Midwifery School of Razi, Kerman University of Medical Sciences, Kerman, Iran; ^2^Nursing Care Research Center, School of Nursing and Midwifery, Iran University of Medical Sciences, Tehran, Iran; ^3^Pediatric and Neonatal Intensive Care Nursing Education Department, School of Nursing & Midwifery, Tehran University of Medical Sciences, Tehran, Iran; ^4^Chamberlain College of Nursing, USA

## Abstract

**Background:**

Happiness and achievement motivation are two important concepts in students' lives. Therefore, this study was aimed at investigating the association between happiness and achievement motivation in undergraduate nursing students and examining their relationship with students' demographic characteristics.

**Method:**

A descriptive correlational study was undertaken; 255 undergraduate nursing students enrolled in one nursing school in an urban area of Iran during the academic year 2017-2018 were included in the study using census method sampling. The demographic data questionnaire, Oxford Happiness Questionnaire (OHQ), and Hermans Achievement Motivation Questionnaire (HAMQ) were used for data collection. Data were analyzed using univariate analysis and multivariate linear regression analysis.

**Results:**

The total mean (SD) of happiness and achievement motivation scores were 40.73 (11.20) and 82.61 (7.50), respectively. There was a direct significant correlation between happiness and achievement motivation (*r* = 0.36, *p* < 0.001). An inverse significant association between happiness and students 21-24 years old (*β* = −3.99, *p* < 0.03) was found. There was a direct significant association between happiness and being married (*β* = 5.33, *p* = 0.03) and interest in nursing (*β* = 4.78, *p* = 0.02). Additionally, achievement motivation was associated directly with female gender (*β* = 1.93, *p* = 0.03) and interest in nursing (*β* = 3.71, *p* = 0.005) and inversely associated with studying in the fourth academic year (*β* = −3.06, *p* = 0.03) and history of course drops (*β* = −3.45, *p* = 0.002).

**Conclusion:**

Our study indicated that there was a direct significant relationship between happiness and achievement motivation in undergraduate nursing students. Therefore, officials and nursing education lecturers should consider programs to increase students' happiness, such as counseling or emotional support programs and workshops.

## 1. Background

Among academic disciplines, nursing plays a vital role in promoting community health, often with other healthcare professionals, so that without skilled nurses, health organizations will not achieve their goals [1]. Preparing nursing graduates with advanced knowledge, skills, and health is the most important mission of nursing schools [2]. Nursing students spend part of their education in the form of practical courses in the hospital and face many stressful conditions along with problems at the university [3]. Previous studies have shown that nursing students experience higher stress than other students in the allied health professions [4]. Therefore, paying attention to their mental health is important [3].

Mental health is an important part of the definition of health that includes all aspects of daily life such as work and school [5]. Mental health has both positive and negative aspects. Negative aspects include problems such as anxiety and depression, and positive aspects include areas such as life satisfaction, resilience, and happiness [6]. Happiness or subjective well-being is one of the basic human needs and is a positive feeling that helps people achieve successful social relationships and their goals [7]. Happiness increases resilience and life satisfaction [8], decreases mistakes, improves performance [9], and can have consequences such as success in marriage, job, relationship, income, health, and longevity [10].

The need for happiness in students seems important [7]. Intercultural research on more than 9,000 students in 47 countries showed that happiness is valued more than all other personal values such as health, love, or wealth [11]. Among nursing students, lack of happiness leads to a decrease in care, self-awareness, endurance, honesty, effort, and love for human beings [12]. In addition, it is believed that happiness in nursing students makes them more willing to help others and improve their physical and mental health, which is important for patient care [13]. In addition to happiness, achievement motivation is an important aspect of student's lives and their education [14]. Achievement motivation as a type of social motivation reflects the desire to strive for success and is a selected factor that affects students' academic achievement, behavior, and memory [15–18]. It is important to pay attention to motivation in educating nursing students [19]. Because the nursing profession deals with people's lives, the lack of motivation can have a devastating effect on public health [20]. Moreover, there is a significant positive relationship between academic achievement motivation and future job commitment in nursing students [21].

Previous studies have shown that happiness and achievement can be related to various variables. For instance, the results of a descriptive-correlational study on 165 students showed that happy students have a better quality of life and higher psychological health [22]. Moltafet et al. conducted a cross-sectional study on 301 students to investigate the relationship between happiness, personality traits, and religious orientation. Based on multiple regression, they reported intrinsic religious orientation (*β* = 0.21) and extroversion (*β* = 0.21) as positive predictors of happiness and neurosis as a negative predictor (*β* = −0.24) [23]. In addition, Paeezi et al. found that people with high levels of happiness had higher academic achievement [24]. In another correlational study on Iranian preuniversity female students, it was found that there was a significant relationship between the self-efficacy of clinical performance and achievement motivation (*r* = 0.61) [25]. Moreover, in an analytical cross-sectional study on 125 students in the second semester, Aramideh et al. reported a significant relationship between achievement motivation score and prayer [26]. It seems that happiness can have an important role in achievement motivation. In addition, achievement motivation helps students to be happy and successful. Especially if they strive for things that make them happy, motivation will cause happiness in their life [9]. Nevertheless, as shown in the literature review and to the best of our knowledge, no study has examined the relationship between happiness and achievement motivation in nursing students. Therefore, this study was conducted to determine the relationship between happiness and achievement motivation in undergraduate nursing students and examine their association with students' demographic characteristics. The hypotheses of the present study were as follows:

- There is a relationship between happiness and achievement motivation in undergraduate nursing students
- There is a relationship between happiness and demographic characteristics of undergraduate nursing students
- There is a relationship between achievement motivation and demographic characteristics of undergraduate nursing students

## 2. Methods

### 2.1. Design and Setting

This was a descriptive, correlational study undertaken on undergraduate nursing students studying in a nursing school in urban Iran during the academic year 2017-2018.

### 2.2. Participants and Sampling

A total of 255 undergraduate nursing students were selected via census method sampling. Participants' eligibility criteria to participate in this study were Iranian nationality, willingness to participate in the study, and lack of mental illness.

The required sample size was estimated at 240 participants based on a similar study [8] with 90% test power and 95% confidence level and with an assumption that the correlation coefficient between happiness and their academic achievement motivation be at least 0.2 for this relationship to be statistically significant.

### 2.3. Instruments

The demographic data questionnaire, Oxford Happiness Questionnaire (OHQ), and Hermans Achievement Motivation Questionnaire (HAMQ) were used in this study for data collection.

#### 2.3.1. Demographic Data Questionnaire

A demographic data questionnaire was designed by the researchers through the review of the literature to examine the relevant demographic variables of participants. The validity of this questionnaire was confirmed using face and content validity methods. This questionnaire included questions about students' gender and age, academic year, marital status, interest in nursing, history of course drops, and residency status.

#### 2.3.2. Oxford Happiness Questionnaire

In the present study, the Oxford Happiness Questionnaire (OHQ) was used to investigate students' happiness. This questionnaire was developed and revised by Hills and Argyle [27]. The questionnaire contains 29 questions with a 4-point Likert scale ranging from 0 = strongly disagree to 3 = strongly agree. The sum of the item scores in this questionnaire ranged from 0 to 87, with a higher score indicating greater happiness [27]. The reliability of the Persian version of the QHQ was confirmed in the students using Cronbach's alpha (*r* = 0.92‐0.93) and the 6-week interval test-retest reliability coefficients (*r* = 73%‐80%) [28, 29].

#### 2.3.3. Hermans Achievement Motivation Questionnaire

The Hermans Achievement Motivation Questionnaire (HAMQ) was applied to assess the achievement motivation in this study. This questionnaire was developed by Hermans [30] in 1970. This is a 29-item self-report questionnaire with a 5-point Likert scale ranging from 1 = lowest motivation to 4 = highest motivation. The obtained scores in this questionnaire vary from 29 to116, in which a score above the average indicates high motivation, and a score below the average indicates low motivation [31, 32]. HAMQ has been used in various studies in Iran, and its reliability and validity have been confirmed [25, 33–35].

### 2.4. Data Collection

Data collection was performed in the academic year 2017-2018 between February and March 2018. The researcher attended the students' classes after the end of class hours to explain the objectives of the research and the application of the results in education to students. Then, the eligibility criteria of students were assessed using face-to-face interviews. Students who were eligible to participate in the study were given three days to complete the informed consent form. Next, the study questionnaires were completed by willing participants. Also, the researcher was available to the participants to answer any questions raised by them during the completion of the questionnaires.

### 2.5. Ethical Considerations

The research proposal was approved by the Ethics Committee of Kerman University of Medical Sciences under the code of IR.KMU.REC.1396.1289.2.5.

### 2.6. Data Analysis

Data were analyzed through descriptive and inferential statistics using the SPSS v.25 software (IBM, Armonk, NY, USA). To describe the characteristics of the participants, frequency (percentage) for categorical variables and mean (standard deviation (SD)) for continuous variables were used. Data were evaluated in view of normal distribution applying the Kolmogorov–Smirnov test and the Shapiro–Wilk test. The mean and standard deviation of happiness and achievement motivation were calculated regarding the normal distribution of the data. The gathered data was evaluated for missing items with no missing data found. Correlation analysis using the Pearson test was used to examine the association between happiness and achievement motivation. In addition, univariate analysis using the independent sample *t*-test or one-way analysis of variance (ANOVA) was conducted to investigate the association between demographic characteristics of participants, happiness, and achievement motivation. Moreover, multivariate linear regression was conducted through STATA software (Version 15, Stata Corporation, College Station, TX, USA) to examine the association between happiness and achievement motivation with demographic characteristics of participants. The assumptions of the linear regression model (independence, linearity, normality, and homoscedasticity) were met, and all independent variables were entered into the regression model. *p* value < 0.05 was set as the level of significance in all analyses.

## 3. Results

All 255 undergraduate nursing students who were studying in the study nursing school during the study period met the eligibility criteria and were included in the study (response rate = 100%). The mean (SD) age of participants was 21.95 (3.6) years old with 55.3% falling into the 18-21-year-old range. The majority of participants were female (60%) and single (89.4%) and had nondormitory residency (57.6%). In addition, 31.8% of participants were studying in the first academic year, 18.4% in the second academic year, 13.8% in the third academic year, and 27.9% in the fourth academic year. Moreover, although most of them were interested in nursing (87.1%), 24.7% had a history of a course drop (Table 1).

The total mean (SD) score of participants' happiness and achievement motivation was reported to be 40.73 (11.20) and 82.61 (7.50), respectively. There was a statistically direct significant correlation between happiness and achievement motivation (*r* = 0.36, *p* < 0.001) (Table 2).

The results of univariate analysis of the association between demographic characteristics of participants and happiness and achievement motivation are presented in Table 3. A statistically significant difference was found between the students' age and their happiness (*p* = 0.005). Based on the post hoc test, students who were 18-21 years old reported higher happiness levels than students who were 21-24 years old (mean difference (MD) = 4.79, *p* = 0.004). In addition, there was a statically significant difference between the students' academic year and their happiness (*p* = 0.005), and according to the post hoc test, students who were in their first academic year reported higher happiness than students in the fourth academic year (MD = 6.33, *p* = 0.003). Moreover, students who had an interest in nursing reported significantly higher happiness (*p* = 0.009).

Female students (*p* = 001), students who had an interest in nursing (*p* = 0.001), and students who had no history of course drops (*p* < 0.001) reported higher achievement motivation. In addition, a statistically significant difference was shown between the students' age and their achievement motivation (*p* = 0.001). According to the post hoc test, students who were 18-21 years old had higher achievement motivation than students who were 21-24 years old (MD = 3.70, *p* < 0.001). Furthermore, there was a statistically significant difference between the students' academic year and their achievement motivation (*p* < 0.001), and based on the post hoc test, students who were in their first academic year reported higher achievement motivation than students in the fourth academic year (MD = 5.70, *p* < 0.001).

The association between happiness and demographic characteristics of the participants is presented in Table 4. Multivariate analyses identified that students' age, marital status, and interest in nursing were significantly associated with happiness (*F* = 6.49; *p* < 0.001). These three variables explained 22% of the total variance in happiness. An inverse significant association was found between happiness and students who were 21-24 years old (*β* = −3.99, 95%confidence interval (CI) = −7.62, -0.35, *p* < 0.03). In addition, there was a direct significant association between happiness and being married (*β* = 5.33, 95%CI = 0.31, 10.35, *p* = 0.03), and interest in nursing (*β* = 4.78, 95%CI = 0.64, 8.92, *p* = 0.02).

Association between achievement motivation and demographic characteristics of the participants is presented in Table 5. According to multivariate analyses, students' gender, academic year, interest in nursing, and history of course drops (*F* = 2.68; *p* = 0.004) were significantly associated with achievement motivation. These four variables explained 10% of the total variance in achievement motivation. A direct association was found between achievement motivation with female gender (*β* = 1.93, 95%CI = 0.10, 3.76, *p* = 0.03) and interest in nursing (*β* = 3.71, 95%CI = 1.11, 6.30, *p* = 0.005). Furthermore, an inverse relationship between achievement motivation and studying in the fourth academic year (*β* = −3.06, 95%CI = −5.85, -0.26, *p* = 0.03), and history of course drops (*β* = −3.45, 95%CI = −5.68, -1.23, *p* = 0.002) was found.

## 4. Discussion

The present study was aimed at investigating the association between happiness and achievement motivation in undergraduate nursing students and examining their relationship with students' demographic characteristics. The results of our study indicated that there was a direct significant association between happiness and achievement motivation. In addition, it was found that there was a statistically significant association between happiness and students' age, being married, and interest in nursing. Moreover, achievement motivation was significantly associated with students' gender, academic year, interest in nursing, and history of course drops. The findings have been discussed with more details through the comparison of similar studies, as follows.

Happiness is a major component of health and one of the main factors of daily human life that has a significant impact on mental health status [36]. In the present study, the mean score of happiness among undergraduate nursing students was below the average (40.73). Near to our findings, previous studies with OHQ reported that happiness scores among nursing students and medical sciences students were 47.84 and 39.95, respectively [37, 38]. However, differences in findings are likely to be influenced by factors including the type of living environment, lifestyle [39], family relationships, and cultural factors [38].

The findings of our study revealed that undergraduate nursing students had high achievement motivation. Similarly, achievement motivation was reportedly high in Swedish nursing students who were studying in all semesters [40]. However, contrary to our findings, the results of a survey study on nursing students from seven hospitals in western China showed that the level of achievement motivation was low [41]. The reasons for explaining this difference may be due to differences in educational courses, dormitory, college environment, studying hours, and the human desire for success [42, 43].

Our study identified a direct significant association between happiness and achievement motivation among nursing students. The researchers did not find any study that investigated the relationship between happiness and achievement motivation in nursing students. Nevertheless, similar to our findings, a prior study found that happiness was directly correlated with achievement motivation among postgraduate students in the humanities [9]. In addition, a recent systematic review identified that happiness improves the students' academic achievement [44]. In explaining the interactions of happiness and achievement motivation, there is a belief that students who have an appropriate level of happiness and peace of mind can demonstrate high motivation achievement, and also high motivation achievement can create more happiness for students [45].

The findings of our study demonstrated that students who were 18-21 years old had higher happiness than those who were 21-24 years old. Consistent with our results, two previous studies reported that there is a significant inverse relationship between age and happiness among medical students [10, 46]. It was shown that the relationship between age and happiness is influenced by socioeconomic status, so that in developing countries, as age increases, happiness declines, while happiness remains stable across the lifespan in developed countries [47].

Based on our results, students' academic year has an inverse relationship with happiness; students in the first academic years were happier than those in the other academic years. Student stress and mental health problems and workload issues are more apparent in nursing programs as the study time progresses because the clinical course of nursing students is gradually increased and they have to spend a lot of time in the hospital in addition to theory courses [48]. Therefore, higher happiness in the first-year nursing students is probably due to fewer assignments they have to complete.

According to the study findings, interest in nursing is a determinant of happiness among nursing students. This finding is in line with the results of a study conducted among college students [49]. Students interested in their field of study strive to develop positive emotions, knowledge, and personal worth. As a result, they likely have a higher level of happiness [50].

The findings of this study indicated a direct significant association between happiness and being married among nursing students. However, two previous studies did not show a significant relationship between happiness and students' marital status [51, 52]. Consistent with our findings, a previous study reported that happiness was higher in married individuals than in single individuals [53]. Marriage can improve the sense of social support and strengthen positive attitudes and feelings, as well as psychological well-being, which can lead to increased happiness [54].

In the present study, a positive association was seen between achievement motivation and female nursing students. Similarly, the findings of a study also showed that the achievement motivation and academic performance of female students were higher than those of males [5]. However, previous studies pointed out that the difference in the achievement motivation between female and male students is not merely affected by gender, and factors such as parents, teachers, student socialization, role modeling, and media may play an important role in this relationship [55–57].

According to our findings, there was a direct association between achievement motivation and interest in nursing. Our findings were similar to the results of a cohort study of nursing students in Norway that found a significant positive relationship between achievement motivation and interest in the field of study [19]. Furthermore, it has been shown that the average academic achievement in high-interest nursing students was higher than that in low-interest students [58]. Interest in the field of study evokes positive feelings about the future and prevention of failure, which can play a role in motivating achievement [59].

Additionally, it was found in this study that students who were 18-21 years old and in their first academic years had higher achievement motivation. In the study by Yilmaz et al., the motivation of first-year nursing students was reported to be high [60]. It seems that the nursing students start their education more willingly [40, 61]. Another study found that the nursing students' motivation to finish their program of study decreased with the number of semesters [61]. The shortage of nursing in the Iranian health system is critical, and even nurses with low grades can be easily employed in the Iranian health system [62]. It seems that nursing students' motivation may decrease with the number of semesters by the thought that nursing is a safe job. In addition, a feeling of the large salary gap between nurses and physicians in Iran [62] could lead to a decrease in the motivation of Iranian nursing students over time.

Lastly, our findings identified an inverse relationship between achievement motivation and the history of courses dropped in nursing students. Kloster's study documented that failing exams reduces students' achievement motivation [63]. Also, a study showed that a student who had failed several courses reported zero scores of motivation [40].

### 4.1. Strengths and Limitations

To the best of our knowledge, the present study was the first to investigate the relationship between happiness and achievement motivation in nursing students, which makes it different from previous studies. Nevertheless, our study had several limitations. Firstly, a cross-sectional design was used in this study; therefore, our results are based on correlations and do not prove causation. Hence, experimental research is suggested in the future to prove the cause-and-effect relationship between happiness and achievement motivation. Secondly, the sample size was small and participants were limited to one medical sciences university in Iran, which can influence the generalization of the results to the other contexts. Therefore, to provide more evidence, future studies are recommended to be conducted on students in different contexts. Thirdly, in the international literature, we did not find a study that examined the relationship between happiness and achievement motivation in nursing students, so it was not possible to fully compare our findings with similar literature. Fourthly, although we adjusted the happiness and achievement motivation for some demographic characteristics, we lacked information on other characteristics such as socioeconomic status which may have led to unmeasured confounding. Lastly, although the criteria for conducting a multivariate linear regression model were met, probable model misspecification could lead to biased coefficients.

## 5. Conclusions

With the increasing development of the world and the creation of new job opportunities, the need for educational readiness and, consequently, academic achievement and achievement motivation in students has become vital. Therefore, it is necessary to pay attention to the factors affecting achievement motivation. Our study indicated that there was a direct significant relationship between happiness and achievement motivation in undergraduate nursing students. Therefore, officials and nursing education lecturers should consider programs to increase students' happiness, such as counseling or emotional support programs and workshops. In addition, it might be useful to consider qualitative methods in future research, i.e., interviewing student nurses to investigate the potential clashes and problems that can arise between clinical and academic nursing. This could provide useful insights into how institutions can improve teaching and, therefore, motivation.

## Figures and Tables

**Table 1:** The participants' characteristics according to demographic and social features (*N* = 255).

Variables		Frequency (%)
Gender	Female	153 (60)
	Male	102 (40)
Age	18-21	141 (55.3)
	21-24	92 (36.1)
	>24	22 (8.6)
Academic year	1	81 (31.8)
	2	47 (18.4)
	3	56 (13.8)
	4	71 (27.9)
Marital status	Single	228 (89.4)
	Married	27 (10.6)
Interest in nursing	Yes	222 (87.1)
	No	33 (12.9)
History of course drops	Yes	63 (24.7)
	No	192 (75.3)
Residency status	Dormitory	108 (42.4)
	Nondormitory	147 (57.6)

**Table 2:** Happiness and achievement motivation and their correlation (*N* = 255).

Variable	Mean	SD	Minimum	Maximum	*r*	*p* value
Happiness	40.73	11.20	11	77	0.36	<0.001
Achievement motivation	82.61	7.50	63	100		

**Table 3:** Univariate analysis of factors associated with achievement motivation and happiness (*N* = 255).

Variables		Happiness mean (SD)	*p* value^a^	Achievement motivation Mean (SD)	*p* value^a^
Gender	Female	40.79 (10.73)	0.96	83.80 (6.82)	0.001
	Male	40.78 (11.96)		80.72 (8.26)	
Age	18-21	42.53 (11.51)	0.005^b^	84.11 (7.33)	0.001^b^
	21-24	37.77 (10.08)		80.41 (7.78)	
	>24	42.45 (11.67)		82.13 (5.74)	
Academic year	1	44 (10.35)	0.005^b^	84.29 (7.37)	<0.001^b^
	2	39.73 (10.96)		83.02 (5.91)	
	3	40.63 (12.79)		84.91 (6.84)	
	4	37.86 (10.27)		78.59 (7.82)	
Marital status	Single	40.43 (11.26)	0.18	82.62 (7.50)	0.90
	Married	43.84 (10.51)		82.44 (8.13)	
Interest in nursing	Yes	41.50 (10.84)	0.009	83.23 (23)	0.001
	No	36.03 (12.61)		78.39 (8.89)	
History of course drops	Yes	39.01 (11.05)	0.13	78.73 (7.99)	<0.001
	No	41.36 (11.24)		83.88 (6.96)	
Residency status	Dormitory	40.72 (10.71)	0.93	83.18 (7.70)	0.28
	Nondormitory	40.83 (11.81)		82.15 (7.45)	

^a^Independent sample *t*-test. ^b^One-way analysis of variance (ANOVA).

**Table 4:** Multivariate linear regression analysis of factors associated with happiness (*N* = 255).

Variables	Unstandardized coefficients *β*	95% CI	Standardized coefficients *β*	*p* value
*Gender*				
Male^†^				
Female	-0.85	-3.76, 2.06	-0.03	0.56
*Age*				
18-21^†^				
21-24	-3.99	-7.62, -0.35	-0.17	0.03
>24	-1.76	-7.80, 4.27	-0.04	0.56
*Academic year*				
1^†^				
2	-3.43	-7.48, 0.61	-0.11	0.09
3	-1.52	-5.58, 2.53	-0.05	0.46
4	-3.30	-7.76, 1.14	-0.13	0.14
*Marital status*				
Single^†^				
Married	5.33	0.31, 10.35	0.14	0.03
*Interest in nursing*				
No^†^				
Yes	4.78	0.64, 8.92	0.14	0.02
*History of course drops*				
No^†^				
Yes	-0.72	-4.26, 2.81	-0.02	0.68
*Residency status*				
Dormitory^†^				
Nondormitory	0.44	-2.36, 3.25	0.01	0.75

^†^Denotes reference category.

**Table 5:** Multivariate linear regression analysis of factors associated with achievement motivation (*N* = 255).

Variables	Unstandardized coefficients *β*	95% CI	Standardized coefficients *β*	*p* value
*Gender*				
Male^†^				
Female	1.93	0.10, 3.76	0.12	0.03
*Age*				
18-21^†^				
21-24	-1.91	-4.19, 0.37	-0.12	0.10
>24	-0.25	-4.04, 3.53	-0.009	0.89
*Academic year*				
1^†^				
2	-0.51	-3.05, 2.02	-0.02	0.68
3	1.83	-0.71, 4.38	0.10	0.15
4	-3.06	-5.85, -0.26	-0.18	0.03
*Marital status*				
Single^†^				
Married	0.22	-2.92, 3.37	0.008	0.88
*Interest in nursing*				
No^†^				
Yes	3.71	1.11, 6.30	0.16	0.005
*History of course drops*				
No^†^				
Yes	-3.45	-5.68, -1.23	-0.19	0.002
*Residency status*				
Dormitory^†^				
Nondormitory	-0.78	-2.54, 0.97	-0.05	0.38

^†^Denotes reference category.

## Data Availability

The data used for this study are available from the corresponding author (MM), upon reasonable request.
